# A liquid-crystal-based DNA biosensor for pathogen detection

**DOI:** 10.1038/srep22676

**Published:** 2016-03-04

**Authors:** Mashooq Khan, Abdur Rahim Khan, Jae-Ho Shin, Soo-Young Park

**Affiliations:** 1 Department of Polymer Science & Engineering, Polymeric Nanomaterials Laboratory, School of Applied Chemical Engineering, Kyungpook National University, #1370 Sangyuk-dong, Buk-gu, Daegu 41566, Korea; 2 School of Applied Biosciences, Kyungpook National University, #1370 Sangyuk-dong, Buk-gu, Daegu 41566, Korea

## Abstract

A liquid-crystal (LC)-filled transmission electron microscopy (TEM) grid cell coated with the cationic surfactant dodecyltrimethylammonium bromide (DTAB), to which a single-stranded deoxyribonucleic acid probe (ssDNA_probe_) was adsorbed at the LC/aqueous interface (TEM_DTAB/DNA_), was applied for the highly specific detection of target DNA molecules. The DTAB-coated E7 (used LC mixture) in the TEM grid (TEM_DTAB_) exhibited a homeotropic orientation, and changed to a planar orientation upon adsorption of the ssDNA_probe_. The TEM_DTAB/DNA_ was then exposed to complementary (target) ssDNA, which resulted in a planar-to-homeotropic configurational change of E7 that could be observed through a polarized optical microscope under crossed polarizers. The optimum adsorption density (2 μM) of ssDNA_probe_ enabled the detection of ≥0.05 nM complementary ssDNA. This TEM_DTAB/DNA_ biosensor could differentiate complementary ssDNA from mismatched ssDNA as well as double-stranded DNA. It also successfully detected the genomic DNAs of the bacterium *Erwinia carotovora* and the fungi *Rhazictonia solani*. Owe to the high specificity, sensitivity, and label-free detection, this biosensor may broaden the applications of LC-based biosensors to pathogen detection.

In recent years, deoxyribonucleic acid (DNA)-based biosensor systems have been developed in response to the growing interests in many fields, such as DNA diagnostics, gene analysis, fast detection of biological warfare agents, specific detection of pathogenic bacteria, and forensic applications^1^. To achieve the desired rapidity, sensitivity, and detection limit, DNA-based pathogen detection schemes often require amplification methods such as the polymerase chain reaction (PCR)^2^. The PCR-based detection methods have been used to identify pathogens by targeting their specific genes. However, these methods rely largely on labor-intensive gel-based detection processes that show poor sensitivity and specificity for the PCR products. Lengthy pre-PCR enrichment is often needed to improve the sensitivity, which adds considerable time to the detection procedure. Alternatively, numerous DNA detection systems based on the hybridization between a DNA target and its complementary probe, which is present either in solution or on a solid support, have been described^3^,^4^,^5^. A variety of DNA hybridization sensors that use various sensing technologies such as fluorescence, electrochemical, surface plasmon resonance, and surface-enhanced Raman scattering methodologies have therefore been proposed^6^,^7^,^8^. Frequently, the detection methods are based on a labeling approach. For example, microarray technologies (which rely on hybridization between DNA sequences on a microarray surface, using semiconductor crystals or quantum dots as fluorescent probes)^9^ are based on nanoparticle-amplified surface plasmon enhanced signals^10^ and the use of redox-active nucleic acid^11^. These homogenous assays allowing determination of DNA are of paramount importance because of their high sensitivity^12^. These techniques use organic fluorescent dyes as direct DNA hybridization probes, which are strongly emissive only when intercalated within the grooves of double-stranded DNA (dsDNA). However, the assays lack sequence specificity, and fluorescence-based techniques with optical systems consisting of laser diode, photodiode, and filter appear to be very costly. The DNA detection systems using traditional energy or electron-transfer pairs for a strand-specific detector^13^ also require chemical labeling of two nucleic acids, or dual modification of the same altered strand. The difficulties in labeling two DNA sites give rise to low yields and singly labeled impurities, which lower the detection sensitivity.

Liquid crystals (LCs) are liquid phases with anisotropic properties. The simplest liquid crystal phase formed by low-molecular-weight rod-like molecules can be seen as a liquid possessing orientational molecular order. The parallel and perpendicular orientations of LC molecules to the surface are referred to as planar and homeotropic, respectively. When observed through polarized optical microscopy (POM) under crossed polarizers, the planar orientation creates birefringence with bright colors, whereas the homeotropic orientation does not allow birefringence and instead creates a black image. LCs have a very low interfacial energy (10^−6^–10^−3^ J/m^2^), causing amplification of the surface-induced ordering to a distance of ~100 μm (10^5^ of molecular length)^14^. These properties, combined with their anisotropic physical properties, allow LCs to amplify and transduce the analyte-to-target binding into an optical output, which can be visualized using POM. Previously, LCs have been used for the detection of chemical and biological species, such as synthetic polymers, simple electrolytes, surfactants^15^,^16^, lipids^17^, proteins^14^,^18^,^19^, and endotoxins^20^. Several studies have also revealed that the response of LCs can be tuned to external stimuli by functionalizing it with surfactants and polyelectrolytes (PEs). For example, the spontaneous adsorption of surfactants and lipids at the LC/aqueous interface induces the planar-to-homeotropic (P-H) configurational change^16^. PEs such as poly(acrylic acid) (PAA)^21^, quaternized poly(4-vinylpyridine) (QP4VP)^22^, poly(dimethylaminoethyl methacrylate) (PDMAEA)^23^, and poly(styrene sulfonate) (PSS)^24^ brushes have been applied to biosensors through adsorption of proteins onto LC-filled transmission electron microscopy (TEM) grid cells. A homeotropic orientation was observed when 5CB was coated with QP4VP (a strong cationic PE) and PSS (a strong anionic PE) regardless of pH, whereas the orientation was found to be pH dependent when it was coated with PAA (a weak anionic PE) and PDMAEA (a weak cationic PE). The pH dependencies of PAA and PDMAEA are due to deprotonation and protonation of the pendant group above and below their pK_a_ values, respectively. A homeotropic-to-planar (H-P) change was observed after the complexation of the PEs with oppositely charged proteins. Thus, the high net charge density on the LC droplet causes a homeotropic orientation, and the H-P change occurs when the net charge density at the LC/aqueous interface decreases as a result of neutralization of the charges through the oppositely charged protein adsorbed. DNA is a strong anionic PE and is known to form insoluble complexes with cationic surfactants in aqueous medium. Previously, the adsorption of single-stranded DNA (ssDNA) to a Langmuir monolayer of a cationic surfactant, as well as the change in molecular area upon hybridization of a target ssDNA (ssDNA_target_) with a membrane-bound probe ssDNA (ssDNA_probe_) at the air/water interface, has been studied^25^.

In this study, as shown in Fig. 1, the cationic surfactant dodecyltrimethylammonium bromide (DTAB) was adsorbed to form a self-assembled monolayer on the surface of an LC-filled TEM grid (TEM_DTAB_). This TEM_DTAB_ grid cell was further functionalized with ssDNA_probe_ (TEM_DTAB/DNA_) by its electrostatic adsorption onto the DTAB monolayer for highly selective hybridization with ssDNA_target_, which could alter the LC anchoring as a result of the altered net charge density at the LC/aqueous interface. The selective detection capability of this TEM_DTAB/DNA_ biosensor was investigated with an ssDNA_target_ as well as mismatched ssDNAs. The biosensor was applied for the detection of pathogens such as bacteria (*Erwinia carotovora*) and fungi (*Rhazictonia solani*). This LC-based biosensor can be used without the need for molecular labeling or sophisticated instruments, giving it a lot of potential for effective applications to the detection of DNA.

## Methods

### Materials

Microscope glass slides (Duran Group, Germany) were cleaned using piranha solution (*caution*: piranha solution is extremely corrosive and must be handled carefully), and subsequently washed with distilled water and dried under nitrogen. Copper TEM specimen grids (G75 with a grid hole width of 285 μm, pitch of 340 μm, bar width of 55 μm, size of 3.05 mm, and thickness of 18 μm) were purchased from Ted Pella, Inc. USA., Octadecyltrichlorosilane (OTS; Sigma Aldrich), E7 (100%; TCI America), DNA from salmon fish (Sigma-Aldrich), and human serum from platelet poor human plasma (Sigma Aldrich) were used as received from the manufacturer. The 16-mer ssDNA sequences 5′-GCACGAAGTTTTTTCT-3′, 5′-CGTGCTTCAAAAAAGA-3′, 5-CGTGCTTCAAATTTCT-3′, 5′-CGTGCTAGTTTTTTCT-3′, and 5′-AACGGGACTCGGGAGA-3′ were purchased from M-Biotech Inc., South Korea. The 19-mer ssDNA sequences 5′-AGAGTTTATCMTGGCTCAG-3′ and 5′-TCCGTAGGTGAACCTGCGG-3′, and the 20-mer ssDNA sequence 5′-TCCTCCGCTTATTGATATGC-3′, were extracted by PCR amplification.

### Isolation of genomic DNA from bacteria

Genomic DNA was isolated from *Erwinia carotovora* (RSC-14 strain) using the protocol of Pitcher *et al*.^26^. The 16 s rRNA gene was amplified in a 50 μL reaction mixture of *Taq* DNA polymerase and universal primers F-27 (5′-AGAGTTTATCMTGGCTCAG-3′) and R-1492 (5′-GRTACCTTGTTACGACTT-3′). The PCR was performed by pre-heating the reaction at 94 °C for 2 min followed by 30 cycles of denaturation at 94 °C for 1 min, annealing at 55 °C for 30 s, extension at 72 °C for 1 min, and a final polymerization at 72 °C for 5 min. The resultant PCR product was purified and stored at −80 °C for further experimentation.

### Isolation of genomic DNA from fungi

Genomic DNA from *Rhazictonia solani* was extracted from lyophilized mycelia according to the cetyltrimethylammonium bromide (CTAB) method, with slight modifications^27^. Briefly, 300 mg of mycelia was thoroughly ground to a fine powder in liquid nitrogen, mixed with 10 mL of extraction buffer (50 mM Tris-HCl [pH 8.0], 50 mM EDTA, 0.7 M NaCl, 2% cetrimide, 1% SDS, and 50 μL β-mercaptoethanol), vortexed thoroughly, and incubated for 30 min at 65 °C with continuous shaking. The lysates were then extracted with an equal volume of chloroform:isoamyl alcohol (24:1) and centrifuged at 12,000 rpm for 10 min at 4 °C. Finally, the DNA was precipitated by mixing with pre-chilled isopropanol and pelleted by centrifugation at 12,000 rpm for 10 min at 4 °C. Subsequently, the DNA was washed twice with 600 μL of 70% ethanol, centrifuged at 12,000 rpm for 1 min, and finally air-dried.

### TEM grid cell preparation

A home-made polydimethylsiloxane flow cell was made using the same method reported elsewhere^19^,^21^. Briefly, glass microscope slides were cleaned and submerged in a solution of toluene (50 mL) and OTS (15 μl) for 1 h at 50 °C. The glass slides were subsequently washed with toluene, acetone, ethanol, and deionized water (DIW) and dried with a stream of nitrogen, and then wrapped in aluminum foil and stored at 40 °C under vacuum for 24 h. The OTS induces the perpendicular alignment of the LC. A copper TEM grid was placed on the surface of the 12 × 8 mm^2^ OTS-coated glass that was glued to another common slide glass with epoxy. A 1 μL drop of E7 was placed on a TEM grid using a 5 μL syringe (Hamilton Co., Reno, Nevada, USA). Excess E7 was removed with a capillary tube to obtain a uniform thin film. The two slide glasses spaced with silicon rubber (2 mm) were then clipped with binder-clips. E7 rather than 4-cyano-4′-pentylbiphenyl (5CB) was used owing to the need to facilitate a high nematic-to-isotropic transition. The inlet and outlet ports for exchanging the solutions were made with needles that were punched through the silicon rubber. The internal volume of the flow cell was 0.4 mL.

### Adsorption of an ssDNA_probe_

The E7-filled TEM grid was exposed to an aqueous solution of DTAB to form a self-assembled monolayer by spontaneous adsorption. In order to optimize the density of DTAB molecules at the E7/aqueous interface, DTAB aqueous solutions of different concentrations (C_DTAB_ = 1–20 mM) were tested. After 30 min, the aqueous solution of an oligonucleotide, 16mer-5′-GCACGAAGTTTTTTCT-3′ (ssDNA_probe_), was injected into the TEM_DTAB_ grid cell and held for 1 h to ensure electrostatic interaction of the probe with the oppositely charged DTAB. In order to obtain a saturated amount of the deposited ssDNA_probe_ on the TEM_DTAB_ grid, different concentrations of the oligonucleotide (C_probe_ = 0.03–5 μM) were tested.

### Detection of an ssDNA_target_

For use as a target DNA (ssDNA_target_), the 5′-CGTGCTTCAAAAAAGA-3′ oligonucleotide was dissolved in DIW. The melting temperature (T_m_, the temperature at which 50% of the nucleotide is annealed) of the ssDNA_target_ to the ssDNA_probe_ was 44.1 °C. The ssDNA_target_ was injected onto the prepared TEM_DTAB/DNA_ biosensor at room temperature and then heated to the T_m_. For analysis of sensitivity, different concentrations of the ssDNA_target_ (C_target_ = 0.05–10 nM) were tested. In order to test the specificity, ssDNA_target_ sequences of different mismatch degrees, such as completely mismatched 5′-AACGGGACTCGGGAGA-3′ (ssDNA_mm_), 3-bp mismatched 5′-CGTGCTAGTTTTTTCT-3′ (ssDNA_3bpmm_), and 2-bp mismatched 5-CGTGCTTCAAATTTCT-3′ (ssDNA_2bpmm_), and dsDNA were tested.

### Preparation of E7_DTAB/DNA_ droplets

E7 (200 μl) was added to 10 mL of an 8 mM aqueous DTAB solution. The mixture solution was vortexed for 30 min to form E7 droplets. The resulting E7_DTAB_ droplets were subsequently washed with DIW and functionalized with a 2-μM ssDNA_probe_ (E7_DTAB/DNA_). E7_DTAB/DNA_ droplets were tested in ssDNA_target_ aqueous solutions at different C_target_s.

### Detection of pathogen DNA

The ssDNA sequences 19mer-5′-AGAGTTTATCMTGGCTCAG-3′ and 19mer-5′-TCCGTAGGTGAACCTGCGG-3′ (or 20mer-5′-TCCTCCGCTTATTGATATGC-3′) were used as probes for the detection of genomic ssDNAs of bacterium (ssDNA_bc_) and fungi (ssDNA_fg_), respectively. The genomic dsDNA from fungi was unzipped to obtain ssDNA_fg_ by incubation at 92 °C for 30 min and quenching with dry ice. For the detection of ssDNA_bc_ and ssDNA_fg_, the solutions were prepared in 5 mM NaCl to minimize the repulsion between the long ssDNA chains and tested at various concentrations (1–5 nM). For analysis of bacterium, an un-degraded ssDNA_bc_ (DNA from PCR) and a thermally degraded ssDNA_bc_ (annealed at 95 °C for 10 min, followed by quenching with dry ice) were used.

### Measurements

Biometra’s TGradient thermal cycler PCR system (Analytik, Jena AG, Germany) was used for genomic DNA extraction. Images of the TEM grid cells during (and after) DNA injection were recorded using POM (ANA-006, Leitz, Germany) under crossed polarizers with a CCD camera (STC-TC83USB, Samwon, Korea). The temperature of the medium was controlled by using a heating glass (70270550, Live Cell Instruments, South Korea) connected to a heating glass controller (CU-201, Live Cell Instruments, South Korea). The optical birefringence (Δn) was measured using a tilting compensator equipped with a calcite compensator plate (type 2073 K, Leitz, Germany), with the source light intensity set to 50% of full illumination. At the 0 position of the compensator, the crystal axis was parallel to the polarizer axis. Δn is defined as Γ/d, where Γ is the phase difference and d is the sample thickness (1.8 × 10^4^ nm). The Γ value was measured from the tilting angle (2i) using the equation 
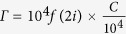
, where 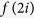 is tabulated in the instrument manual and c is the compensator constant (4.54 × 10^4^). The grey-scale intensity (GI) of the images was measured from 24,800 pixels at a fixed region using Adobe Photoshop CS5. The E7_DTAB/DNA_ droplets were prepared by a vortex mixer (VM-10, DAIHAN Scientific Co., Ltd. South Korea). The charge density on the E7 droplets was studied using zeta potentiometry (Zatasizer Nano ZS90, Malvern Instruments Ltd., UK). All experiments were performed in triplicate and the standard deviations were measured.

## Results and Discussion

### TEM_DTAB/DNA_ preparation

**Figure 2:**
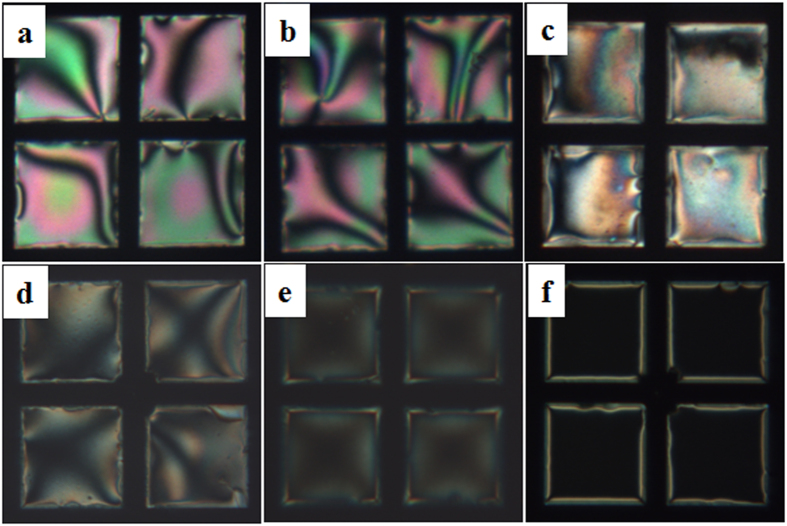
Polarized optical microscopy images of the TEM_DTAB_s at dodecyltrimethylammonium bromide (DTAB) concentrations of (**a**) 0, (**b**) 1, (**c**) 2, (**d**) 4, (**e**) 7, and (**f**) 8 mM. TEM_DTAB_s, liquid-crystal-filled transmission electron microscopy grids coated with DTAB.

Figure 2 shows the POM images of the TEM_DTAB_s at different C_DTAB_s in aqueous medium. The E7 exhibited a planar orientation at C_DTAB_ = 0 (Fig. 2a) that was maintained until C_DTAB_ = 1 mM (Fig. 2b). Dark regions appeared and grew with an increase in C_DTAB_ (2, 4, and 7 mM), as shown in Fig. 2c–e. A clear homeotropic orientation was observed at C_DTAB_ ≥ 8 mM (Fig. 2f). The homeotropic orientation was due to the adsorption of the hydrophobic tail of the surfactant into the E7 molecules. Past studies have shown that the interaction of the surfactant tail and LC molecules largely dictates the orientation of the LC^28^,^29^. For example, compression of the Langmuir monolayer of 4-octyl-4′-cyanobiphenyl (8CB) and pentadecanoic acid (PDA) on water resulted in the vertical alignment of 8CB^16^. This alignment of 8CB was attributed to the interaction between the 8CB molecules and the tail of PDA, and the surfactant concentration had to be high enough to achieve a homeotropic orientation. Similarly, the self-assembled monolayers of surfactants deposited on the LC surface were found to control the orientation of the underneath LC^30^. The 8 mM solution of DTAB caused a clear homeotropic orientation of E7 at the LC/aqueous interface, and so the concentration of DTAB in water was fixed at 8 mM for all experiments, unless otherwise specified.

**Figure 3:**
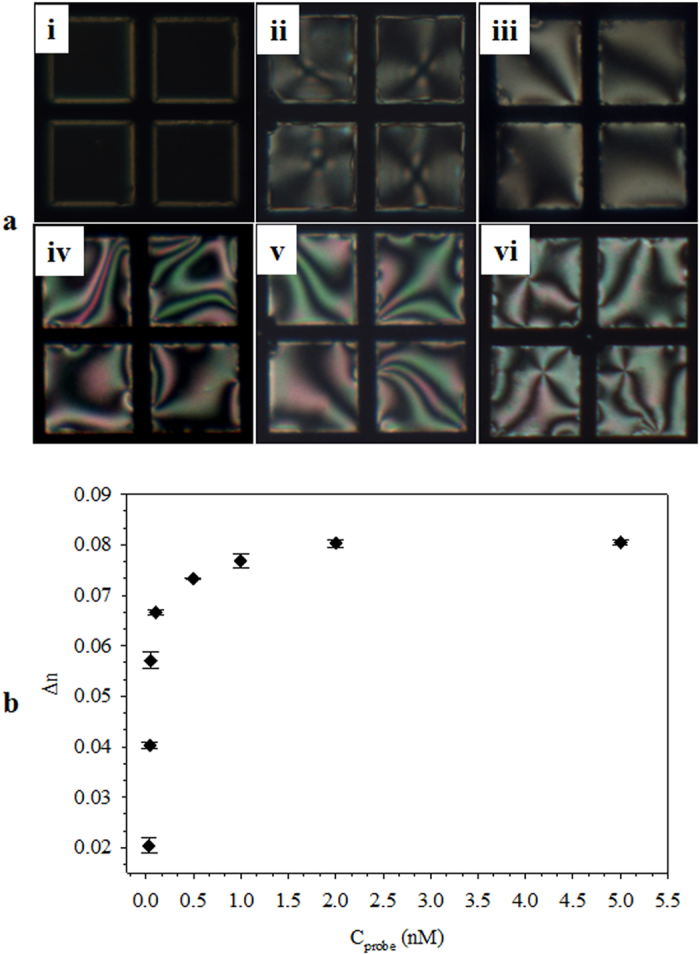
(**a**) Polarized optical microscopy images of TEM_DTAB/DNA_s at ssDNA_probe_ concentrations (C_probe_) of (i) 0.03, (ii) 0.050, (iii) 0.100, (iv) 0.500, (v) 1, and (vi) 2 μM. (**b**) The measured birefringence (Δn) of (a) as a function of C_probe_. The error bars represent standard deviation. TEM_DTAB/DNA_s, liquid-crystal-filled transmission electron microscopy grids coated with dodecyltrimethylammonium bromide (DTAB) and ssDNA_probe_.

In order to obtain the optimum ssDNA_probe_, the TEM_DTAB/DNA_ grids were prepared at different C_probe_ (16mer) values. The homeotropic orientation was maintained at C_probe_ = 0.03 μM (Fig. 3ai) but slowly changed to a planar orientation with slight birefringence at C_probe_ = 0.05 μM (Fig. 3aii). The birefringence became more visible at C_probe_ = 0.1 and 0.5 μM (Fig. 3aiii,iv) and was more evident at C_probe_ ≥1 μM (Fig. 3av,vi). The H-P change was due to electrostatic interaction between the surfactant and ssDNA_probe_. The complexation of Langmuir monolayers of cationic surfactants of CTAB and octadecylamine with ssDNA has been studied by Symietz *et al*.^25^. It was found that the interfacial electrostatic interactions between the surfactants and ssDNA resulted in an effective increase in the size of the surfactant head because of steric interactions or intercalation of ssDNA into the head-group layer. Other studies have reported that the high charge density on the LC surface by decoration of PE causes homeotropic orientation of the LCs at the LC/aqueous interface, whereas neutralizing the charge density with oppositely charged proteins results in H-P transition of the LC molecules^23^,^24^. The birefringence (Δn) is an indicator of the degree of angle tilt of the LC molecules^31^. The charge on the LC surface may affect the tilting angle of LC molecules against the surface. Figure 3b shows the plot of Δn against C_probe_. The Δn increased rapidly until C_probe_ = 2 μM and then became saturated after that. The Δn values obtained for 2 μM of 19-mer and 20mer ssDNA_probe_s were 0.079 ± 0.005 and 0.08 ± 0.007, respectively, these were consistent with the value of 16-mer. The increase of Δn was because the initial perpendicular orientation of LC was progressively tilted to the parallel orientation as C_probe_ increased until reaching 2 μM. Further increase in C_probe_ did not change the LC orientation, and so 2 μM ssDNA_probe_ was used for preparation of the TEM_DTAB/DNA_, unless otherwise specified.

### Detection of target DNA

**Figure 4:**
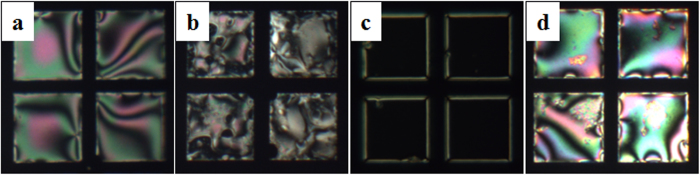
Polarized optical microscopy images of the TEM_DTAB/DNA_s after injection of a 50 nM aqueous solution of ssDNA_target_ into the cell at (**a**) room temperature, (**b**) immediately, and (**c**) 5 min after the temperature reached T_m_, and (**d)** at T_m_ in the absence of the ssDNA_target_. TEM_DTAB/DNA_s, liquid-crystal-filled transmission electron microscopy grids coated with dodecyltrimethylammonium bromide (DTAB) and ssDNA_probe_.

The initial planar orientation of the TEM_DTAB/DNA_ was preserved (Fig. 4a) after injection of a 50 nM aqueous solution of ssDNA_target_ into the cell at room temperature. As the temperature was increased to the T_m_ of ssDNA_target_ (44.1 °C), homeotropic domains started to appear (Fig. 4b) and covered the entire area, making it completely dark 5 min after injection of the ssDNA_target_ (Fig. 4c). This P-H transition is due to the hybridization between the ssDNA_probe_ and ssDNA_target_, which increases the net charge density at the LC/aqueous interface. The PCR, uses a high concentration of counter ions (NaCl 0.5–1 M) to minimize the repulsion between the ssDNA chains and promote hybridization. However, the oppositely charged cationic surfactant bound with the ssDNA_probe_ provided a favorable environment for hybridization without salt. Since an increase in temperature may affect the LC orientation, the TEM_DTAB/DNA_ was heated at T_m_ (44.1 °C) in order to confirm that the homeotropic orientation was due to the hybridization only. No P-H change was observed (Fig. 4d), indicating that the hybridization of the ssDNA_probe_ and ssDNA_target_ caused the orientation change of E7 at the LC/aqueous interface.

### Sensitivity and mechanism of detection

**Figure 5:**
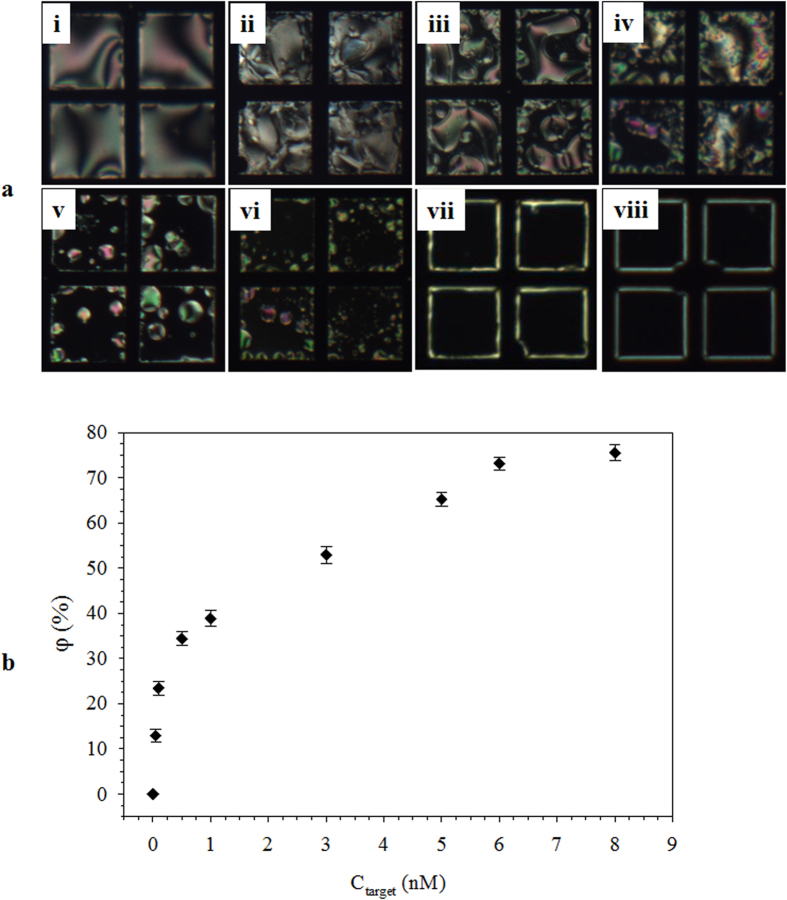
(**a**) Polarized optical microscopy images of TEM_DTAB/DNA_s at ssDNA_target_ concentrations (C_target_) of (i) 0.05, (ii) 0.1, (iii) 0.5, (iv) 1, (v) 3, (vi) 5, (vii) 6, and (viii) 8 nM. (**b**) The loss of brightness (φ) as a function of C_target_. The error bars represent standard deviation. TEM_DTAB/DNA_s, liquid-crystal-filled transmission electron microscopy grids coated with dodecyltrimethylammonium bromide (DTAB) and ssDNA_probe_.

The performance of TEM_DTAB/DNA_ was tested at different C_target_ values. At C_target_ <0.05 nM, no P-H change was observed. At C_target_ = 0.05 nM, nucleation of the homeotropic domain started to become evident (Fig. 5ai). At 0.1 ≤ C_target_ ≤5 nM, the homeotropic domain grew with the increase in C_target_ (Fig. 5aii–vi). At C_target_ ≥ 6 nM, clear homeotropic patterns were observed (Fig. 5avii,viii). These results indicate that even small amounts of ssDNA_target_ could significantly alter the E7 orientation. The increase in loss of brightness (φ) concurrent with progressive P-H change from the increase in C_target_ is an indication of the quantity of the hybridized ssDNA_target_. The φ was calculated using Equation (1):


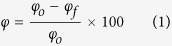


where φ_o_ and φ_f_ are the brightness of the TEM_DTAB/DNA_ grid area before and after the interaction with ssDNA_target_, respectively, where the brightness was measured from the GI value. Figure 5b shows the plot of φ as a function of C_target_. The φ increased sharply at C_target_ ≤ 0.1 nM, and then slowly increased until C_target_ = 6 nM, and became saturated after that. A notable difference in the POM image of the TEM_DTAB/DNA_ grid area and the φ value can be found at C_target_ values of 0.04 and 0.05 nM, respectively. For the detection of DNA, Tan *et al*. used an LC biosensing technique that was based on the use of biotinylated DNA and ascorbic acid-2-phosphate bonded with streptavidin, followed by enzymatic silver metal deposition^32^. This system could detect up to 0.001 nM of DNA and saturation was at 0.1 nM. However, the use of labeled DNA and enzyme makes this method complex and costly for practical application. Our TEM_DTAB/DNA_ biosensor is a comparatively simpler and label-free technique that can detect DNA_target_ at as low as 0.05 nM concentration, with a large saturation value of 6 nM.

**Figure 6:**
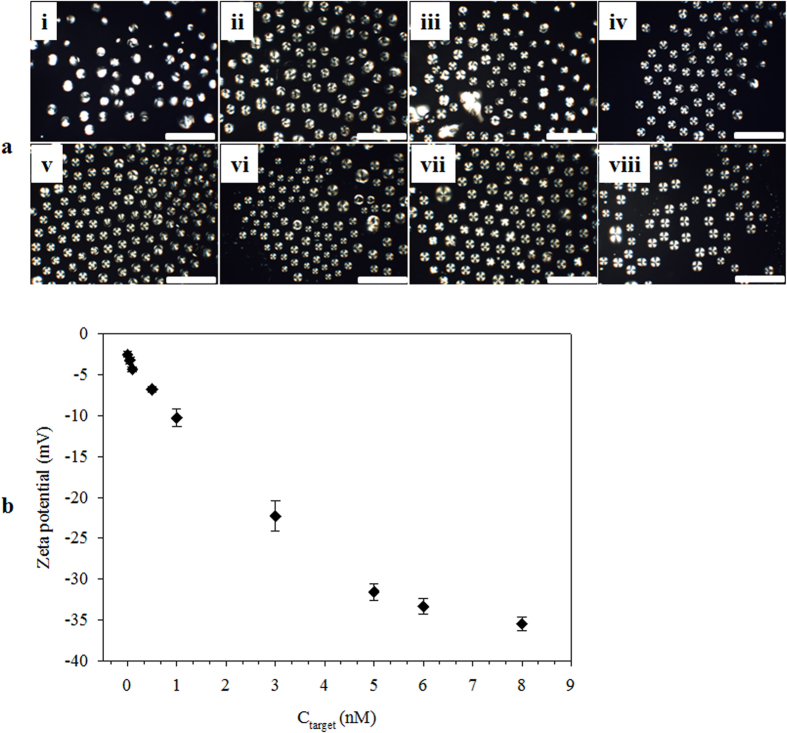
(**a**) Polarized optical microscopy images of the E7_DTAB/DNA_s droplets at ssDNA_target_ concentrations (C_target_) of (i) 0, (ii) 0.1, (iii) 0.5, (iv) 1, (v) 3, (vi) 5, (vii) 6, and (viii) 8 nM. The scale bars are 50 μm. (**b**) Zeta potential as a function of C_target_. The error bars represent standard deviation. E7_DTAB/DNA_s, E7 droplets coated with dodecyltrimethylammonium bromide (DTAB) and ssDNA_probe_.

Charge states of the TEM_DTAB/DNA_ at different C_target_ values were determined using the E7_DTAB/DNA_ droplets. The E7_DTAB/DNA_ droplets at different C_target_s was observed at T_m_ = 44.1 °C under cross polarizers (Fig. 6a), and their net charge density was found by zeta potential (Fig. 6b). A similar bipolar to radial orientational change (B-R change) was observed with an increase of C_target_. This B-R change was attributed to the hybridized ssDNA_target_, which increased the net charge density at the E7/aqueous interface. The zeta potential of the E7_DTAB/DNA_ droplets confirms that the anionic charge density of the E7_DTAB/DNA_ droplet increased with an increase of C_target_, reaching a saturation value of −32 mV at a C_target_ of 5 nM. Compared with φ, the C_target_ at which the saturation occurs (5 nM) during zeta potential measurements differs from that of GI measurements (6 nM). This difference may be due to a difference in the experimental platform. Moreover, these results support our claim that hybridization of ssDNA_target_ causes high anionic charge density leading to an orientational change of the liquid crystal in TEM_DTAB/DNA_.

### Selectivity and stability

**Figure 7:**
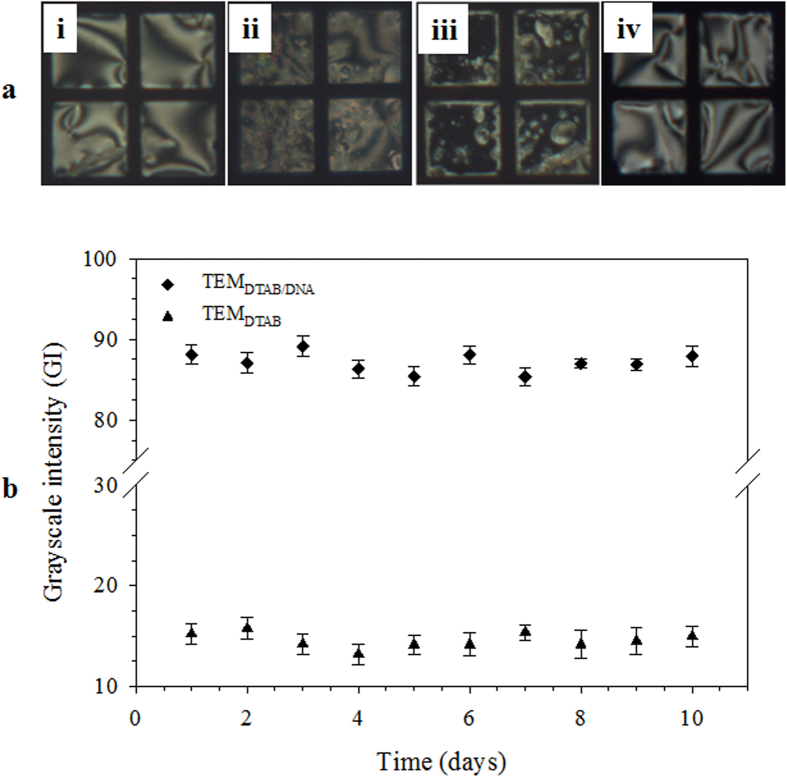
(**a**) Polarized optical microscopy images of TEM_DTAB/DNA_s in 10 nM solutions of (i) complete, (ii) 3-bp, and (iii) 2-bp mismatched ssDNAs, and (iv) dsDNA, and (**b**) GI as a function of time. The error bars represent standard deviation. TEM_DTAB/DNA_s, liquid-crystal-filled transmission electron microscopy grids coated with dodecyltrimethylammonium bromide (DTAB) and ssDNA_probe_.

In order to determine the selectivity of TEM_DTAB/DNA_, ssDNAs with different degrees of mismatch were tested. The TEM_DTAB/DNA_s was subjected to 10 nM aqueous solutions of each ssDNA_mm_, ssDNA_3bpmm_, and ssDNA_2bpmm_. Whereas there was no P-H change in the ssDNA_mm_ solution (Fig. 7ai), the ssDNA_3bpmm_ solution generated scattered homeotropic domains (Fig. 7aii) and the ssDNA_2bpmm_ solution exhibited dominant homeotropic domains (Fig. 7aiii). Thus, ssDNA with complete mismatch (ssDNA_mm_) does not change the initial planar orientation owing to difficulty in hybridization with ssDNA_probe_, but ssDNAs with 2- and 3-bp mismatches (DNA_2bpmm_ and DNA_3bpmm_) cause scattered homeotropic domains, indicating that the degree of mismatch in ssDNA_target_ influences the LC configuration through different degrees of hybridization. The specificity for the target ssDNA is known to decrease as the chain length of the probe ssDNA decreases^33^. However, this TEM grid biosensor differentiated complete, 2-bp, and 3-bp mismatched ssDNAs from a complementary (target) ssDNA, even in 16-mer oligonucleotides, indicating that it provides high specificity. In order to check whether the hybridization between target and probe ssDNAs is a main factor for changing the LC orientation, dsDNA (which does not have the ability for hybridization) was tested. The planar orientation was preserved (Fig. 7aiv), confirming that the P-H change is due to the hybridization between the probe and target ssDNAs. The activity of TEM_DTAB/DNA_ biosensor was mainly dependent on the stability of the adsorbed DTAB and ssDNA_probe_. The leaching of DTAB or ssDNA in aqueous medium may result in change in the optical appearance of the E7 and the GI. The GI vs. time in days was found from various TEM_DTAB_ and TEM_DTAB/DNA_ grid cells stored at room temperature as shown in Fig. 7b. The TEM_DTAB/DNA_ grid cells were tested for ssDNA_target_ (8 nM) detection with the passage of time. Clear homeotropic orientations (Figure supporting information (SI) 1) and no significant change in GI (Fig. 7b) over 10 days suggested that it could detect ssDNA_target_ in the same manner as the freshly prepared TEM_DTAB/DNA_ grid cell. Thus these results reflect adequate stability of the TEM_DTAB/DNA_ grid biosensor.

### Detection of pathogens and real sample analysis

**Figure 8:**
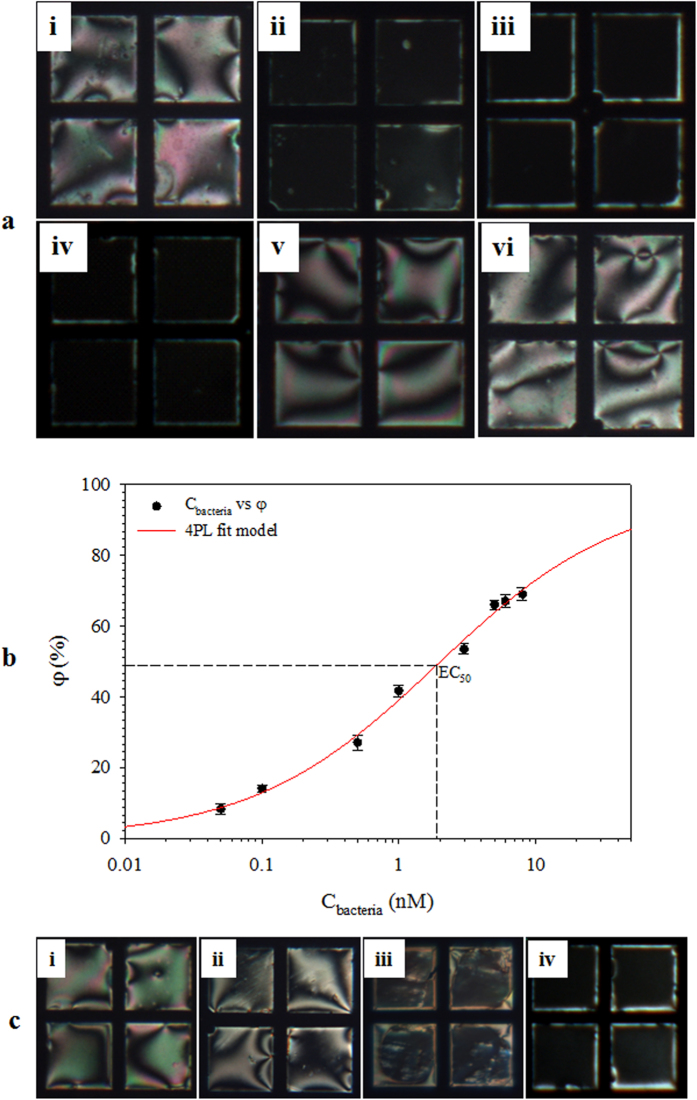
(**a**) Polarized optical microscopy images of TEM_DTAB/DNA_s at T_m_ = 52.4 °C, functionalized with F27 in 5 mM NaCl solutions of (i) ssDNA_bc_ and (ii) thermally degraded ssDNA_bc_; with (iii) ITS1 and (iv) ITS 4 in a 5 mM NaCl solution of ssDNA_fg_; with (v) ITS1 in a 10 nM dsDNA_fg_ solution; and with (vi) a random 16-mer 5′-AACGGGACTCGGGAGA-3′ oligomer in a 5 mM NaCl solution of the thermally degraded ssDNA_bc_. (**b**) The loss of brightness (φ) as a function of C_bacteria_, the error bars represent standard deviation and (**c**) TEM_DTAB/DNA_ in a mixture solution of human serum with ssDNA_bc_ contents of (i) 0, (ii) 0.5, (iii) 3, and (iv) 6 nM. F27, ssDNA oligomer complementary to *Erwinia carotovora* DNA; ITS1/ITS4, universal primers; ssDNA_bc_, genomic DNA of *Erwinia carotovora*; ssDNA_fg_; genomic DNA of *Rhazictonia solani*; TEM_DTAB/DNA_s, liquid-crystal-filled transmission electron microscopy grids coated with dodecyltrimethylammonium bromide (DTAB) and ssDNA_probe_; T_m_, temperature at which 50% of the nucleotide anneals.

The TEM_DTAB/DNA_ grid cell was used for the detection of genomic DNAs of the pathogens *Erwinia carotovora* and *Rhazictonia solani* (ssDNA_bc_ and ssDNA_fg_, respectively). *Erwinia carotovora* is a gram-negative bacterium carries the specific genomic DNA sequence 5′-TCTCAAACTAGAACCGAGTC-3′ in its DNA chain, the complementary ssDNA oligomer 5′-AGAGTTTATCMTGGCTCAG-3′ (F27) was used as the detection probe. Similarly, because the *Rhazictonia solani* fungi has DNA sequences complementary to the universal primers ITS1 (5′-TCCGTAGGTGAACCTGCGG-3′) and ITS4 (5′-TCCTCCGCTTATTGATATGC-3′), either of these primers can be used as the detection probe. The response of TEM_DTAB/DNA_s functionalized with F27 was observed in a 10 nM aqueous ssDNA_bc_ solution. The planar orientation was maintained when the temperature was increased and held at T_m_ = 52.4 °C (Fig. 8ai). However, clear homeotropic domains appeared 5 min after the thermally degraded ssDNA_bc_ was injected (Fig. 8aii), indicating that the high-molecular-weight target ssDNA had difficulties in hybridizing with the ssDNA_probe_, consistent with other reports^33^. Figure 8aiii,iv show the POM images of TEM_DTAB/DNA_s functionalized with ITS1 and ITS4, respectively, after injection of the thermally unzipped ssDNA_fg_ into the TEM_DTAB/DNA_ cell. Clear homeotropic orientations were observed for both primers. However, no P-H change was observed when the dsDNA solution of *Rhazictonia solani* fungus was injected (Fig. 8av), indicating again that hybridization was the key factor for this orientational change. In order to confirm whether hybridization is the key factor or not, a random 16-mer 5′-AACGGGACTCGGGAGA-3′ ssDNA was used as a probe ssDNA, with otherwise same experimental conditions. The planar orientation was preserved when the thermally degraded ssDNA_bc_ (Fig. 8avi) solution was injected, indicating that the P-H change was due to hybridization. Thus, this TEM_DTAB/DNA_ biosensor has the capability to be used as a biosensor for the specific detection of pathogens.

The half maximal effective concentration (EC_50_, the concentration of an analyte half way between the baseline and maximum) evaluates the suitability and the performance of an assay. A common way of defining the EC_50_ response is to use a mathematical model (eq. (2)) known as a 4-parameter logistic model.


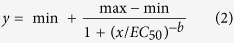


where y, x, min, max, and b are the response, concentration, lower asymptote, upper asymptote, and hillslope, respectively. The response of TEM_DTAB/DNA_ was observed at different concentrations of ssDNA_bc_ (C_bacteria_) as shown in Figure SI2. The φ increased with an increase in C_bacteria_ (Fig. 8b). The estimated EC_50_ of 1.89 nM was obtained, corresponding to the min and max values of 0.08 and 97%, respectively with b = 0.64. This value may be slightly higher than the actual value because of the missing C_bacteria_ on the upper plateau as reported elsewhere^34^. However, this EC_50_ value was within the linear range (0.5–6 nM) of the TEM_DTAB/DNA_ biosensor. In order to find the effect of associated proteins and other active chemicals on ssDNA_bc_ detection, mixture solutions of human serum (2 mg/mL) and thermally degraded ssDNA_bc_ (C_bacteria_ = 0.5, 3, and 6 nM) were prepared. The human serum consists of cholesterol, glucose, sodium, iron, proteins, endotoxin, and triglyceride. The TEM_DTAB/DNA_ maintained the planar orientation in aqueous solution of human serum (Fig. 8ci). A slight P-H change was observed at C_bacteria_ = 0.5 nM (Fig. 8cii). The homeotropic orientation became more visible at high C_bacteria_ = 3 and 6 nM (Fig. 8ciii,iv). The φ values for the mixture solutions were found to be 28.32 ± 1.52, 47.69 ± 1.89, and 68.13 ± 1.97 which lies in the standard error range of the corresponding pure solutions 27.03 ± 1.38, 53.53 ± 1.80, and 66.98 ± 1.54 at C_bacteria_ = 0.5, 3, and 6 nM, respectively. Thus, TEM_DTAB/DNA_ provides enough specificity for ssDNA_bc_ detection even in a complex mixture.

## Conclusions

An E7-filled TEM grid cell, functionalized by coating with the cationic surfactant DTAB and subsequent adsorption of ssDNA_probe_ at the LC/aqueous interface, was used for highly specific detection of target DNA molecules. Functionalization of the DTAB-coated TEM grid cell was successfully achieved by the electrostatic interactions between the cationic DTAB and the anionic ssDNA_probe_. The changes in the LC orientation brought about by hybridization between the complementary probe and target ssDNAs in the TEM grid cell could be applied as a biosensor for specific ssDNA_target_ detection, with a sensitivity of 0.05 nM. The P-H change of the LC molecules could be monitored from its optical appearance through POM, without the need for sophisticated instruments. This biosensor can also differentiate mismatched ssDNA from complementary ssDNA, making it suitable for label-free detection of specific pathogen DNA.

## Additional Information

**How to cite this article**: Khan, M. *et al*. A liquid-crystal-based DNA biosensor for pathogen detection. *Sci. Rep.* **6**, 22676; doi: 10.1038/srep22676 (2016).

## Supplementary Material

Supplementary Information

## Figures and Tables

**Figure 1:**
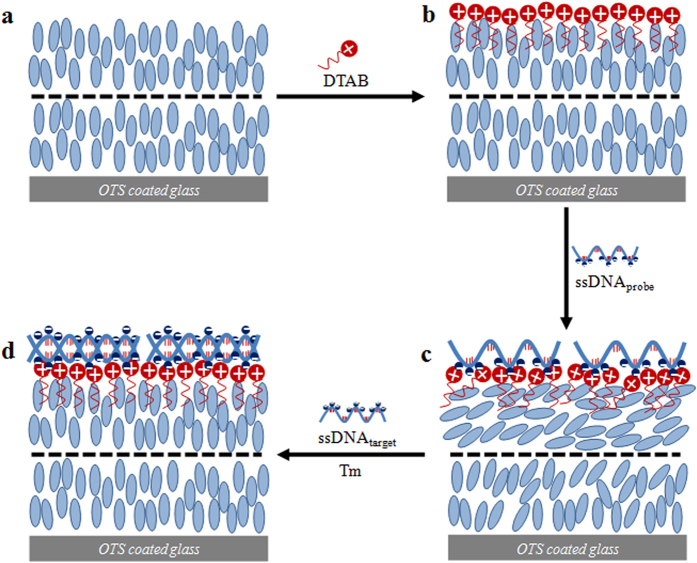
Stepwise assembly of the TEM_DTAB/DNA_: (**a**) E7 filled in a TEM grid placed on the OTS-coated glass, (**b**) TEM_DTAB_, (**c**) TEM_DTAB/DNA_, and (**d**) after hybridization of ssDNA_probe_ with ssDNA_target_. OTS, octadecyltrichlorosilane; TEM_DTAB/DNA_, liquid-crystal-filled transmission electron microscopy grid coated with dodecyltrimethylammonium bromide (DTAB) and ssDNA probe.
