# Corrigendum: A liquid-crystal-based DNA biosensor for pathogen detection

**DOI:** 10.1038/srep46972

**Published:** 2018-05-17

**Authors:** Mashooq Khan, Abdur Rahim Khan, Jae-Ho Shin, Soo-Young Park

Scientific Reports **6**: Article number: 2267610.1038/srep22676; published online: 03 04 2016; updated: 05 17 2018

In this Article, the authors neglected to cite related relevant studies investigating the detection of target DNA at LC/aqueous interface. These references should have been listed in the original reference list as Ref 35-38 and should appear in the text as follows.

In the Introduction section,

“Alternatively, numerous DNA detection systems based on the hybridization between a DNA target and its complementary probe, which is present either in solution or on a solid support, have been described^3-5^.”

should read:

“Alternatively, numerous DNA detection systems based on the hybridization between a DNA target and its complementary probe, which is present either in solution or on a solid support, have been described^3-5,35-38^.”

In the Methods Section,

“The 16-mer ssDNA sequences 5′-GCACGAAGTTTTTTCT-3′, 5′-CGTGCTTCAAAAAAGA-3′, 5-CGTGCTTCAAATTTCT-3′, 5′-CGTGCTAGTTTTTTCT-3′, and 5′-AACGGGACTCGGGAGA-3′ were purchased from M-Biotech Inc., South Korea.”

should read:

“The 16-mer ssDNA sequences 5′-GCACGAAGTTTTTTCT-3′, 5′-CGTGCTTCAAAAAAGA-3′, 5-CGTGCTTCAAATTTCT-3′, 5′-CGTGCTAGTTTTTTCT-3′, and 5′-AACGGGACTCGGGAGA-3′^35^ were purchased from M-Biotech Inc., South Korea.”

These Ref 35–38 are listed below as Ref 1,2,3,4.
