# Supplementary material for: A liquid-crystal-based DNA biosensor for pathogen detection
Source: Sci Rep. 2016 Mar 4;6:22676. doi: 10.1038/srep22676 (PMC4778015; doi:10.1038/srep22676)
Supplement: Supplementary Information [file srep22676-s1.pdf]

Supporting Information

A liquid-crystal-based DNA biosensor for pathogen detection

Mashooq Khan[1], Abdur Rahim Khan[2], Jae-Ho Shin[2] and Soo-Young Park[1]*

[1]Department of Polymer Science & Engineering, Polymeric Nanomaterials Laboratory, School of Applied Chemical Engineering, Kyungpook National University, #1370 Sangyuk-dong, Buk-gu, Daegu 41566, Korea

[2]School of Applied Biosciences, Kyungpook National University, #1370 Sangyuk-dong, Buk-gu, Daegu 41566, Korea

*Correspondence author contact: psy@knu.ac.kr

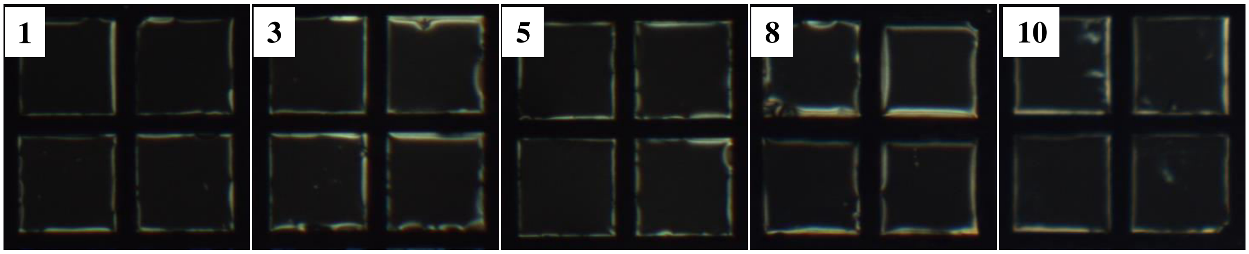

Figure SI 1. Polarized optical microscopy images of $TEM_{DTAB/DNA}$ in 8 nM $ssDNA_{target}$ at time pass in days. The numeral in the inset represents the number of days.

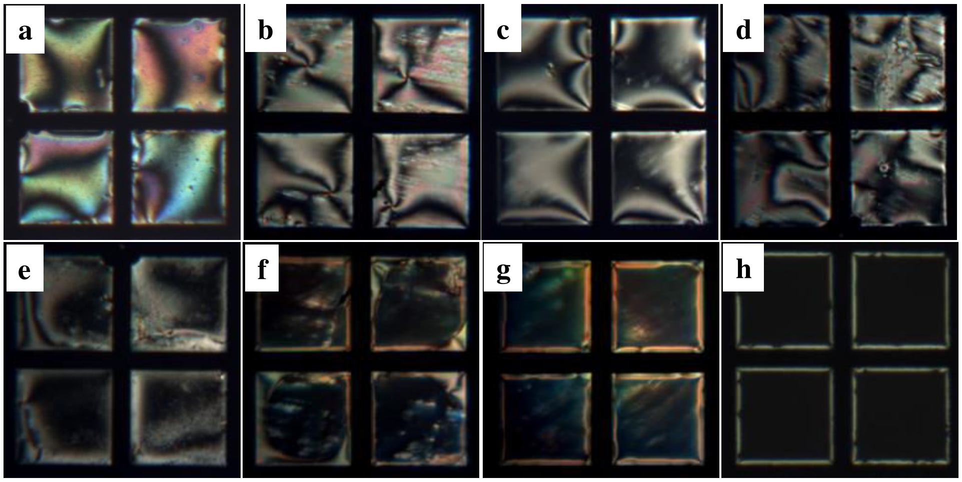

Figure SI 2. Polarized optical microscopy images of $TEM_{DTAB/DNA}$ at $ssDNA_{bc}$ concentrations ($C_{bacteria}$) of (a) 0.05, (b) 0.1, (c) 0.5, (d) 1, (e) 3, (f) 5, (g) 6, and (h) 8 nM.
